# Resistance Determinants and Their Association with Different Transposons in the Antibiotic-Resistant *Streptococcus pneumoniae*

**DOI:** 10.1155/2015/836496

**Published:** 2015-03-26

**Authors:** Izabela Korona-Glowniak, Radoslaw Siwiec, Anna Malm

**Affiliations:** Department of Pharmaceutical Microbiology, Medical University of Lublin, Chodzki 1, 20-093 Lublin, Poland

## Abstract

Multiple resistance of *Streptococcus pneumoniae* is generally associated with their unique recombination-mediated genetic plasticity and possessing the mobile genetic elements. The aim of our study was to detect antibiotic resistance determinants and conjugative transposons in 138 antibiotic-resistant pneumococcal strains isolated from nasopharynx of healthy young children from Lublin, Poland. These strains resistant to tetracycline and/or to chloramphenicol/erythromycin/clindamycin were tested by PCR using the specific genes as markers. The presence of Tn*916* family transposons, carrying *tet*(M) and *int/xis*Tn*916*, was observed in all of the tested strains. Tn*916* was detected in 16 strains resistant only to tetracycline. Tn*6002* and Tn*3872*-related element were found among 99 *erm*(B)-carrying strains (83.8% and 3.0%, resp.). Eight strains harbouring *mef*(E) and *erm*(B) genes were detected, suggesting the presence of Tn*2010* and Tn*2017* transposons. Among 101 chloramphenicol-resistant strains, two variants of Tn*5252*-related transposon were distinguished depending on the presence of *int/xis5252* genes specific for *cat* gene-containing Tn*5252* (75.2% of strains) or *int*_Sp23FST81_ gene, specific for *cat*-containing ICESp23FST81 element (24.8% of strains). In 6 strains Tn*916*-like and Tn*5252*-like elements formed a Tn*5253*-like structure. Besides clonal dissemination of resistant strains of pneumococci in the population, horizontal transfer of conjugative transposons is an important factor of the high prevalence of antibiotic resistance.

## 1. Introduction

Antimicrobial resistance among* Streptococcus pneumoniae* has spread worldwide and it causes higher risk of treatment failure in pneumococcal infections.* S. pneumoniae* is an important human pathogen associated with respiratory tract infections, bacteriemia, and meningitis. Primarily as a commensal, the pneumococcus colonizes the nasopharynx of 10–20% of healthy adults and 40–77% of healthy children [1]. Nasopharyngeal carriage in healthy children is a major factor in the horizontal transmission of pneumococcal strains, especially between children attending day care centers (DCCs) or to other family members, and may be also the source of infection in other individuals. Moreover, pneumococcal nasopharyngeal isolates reflect the strains currently circulating in the community [1, 2].

Multiple resistance of pneumococci especially resistance to macrolides and tetracyclines as well as to chloramphenicol is generally associated with their unique recombination-mediated genetic plasticity and possessing the mobile genetic elements [3, 4]. Two major mechanisms of macrolide resistance in* S. pneumoniae* are noted. The first one is target site modification by a ribosome methylase, encoded by* erm(B)* gene and related to high-level resistance to macrolide, lincosamides, and streptogramin B (MLS_B_ phenotype).* Erm*(B) resistance can be expressed by pneumococci either constitutively (cMLS_B_ phenotype) or inducibly (iMLS_B_ phenotype) [5]. The majority of macrolide-resistant* S. pneumoniae* strains are also resistant to tetracycline. This association is due to the insertion of* erm*(B) into conjugative and composite transposons of the Tn916 family that harbours* tet(M)* gene, encoding ribosome protection proteins. Members of this family, which carry* erm*(B), include Tn*6002*, Tn*1545* (which also carries the kanamycin resistance gene* aph3-III*), and Tn*3872* (which also carries transposase genes* tnpA* and* tnpR*) [6–9].

The second macrolide resistance mechanism is an efflux pump (encoded by* mef* genes) which confers resistance to 14- and 15-member macrolides only (M phenotype). The two main subclasses of* mef* gene in pneumococci are carried on different but related elements:* mef*(A) on the defective transposons Tn*1207.1* or Tn*1207*.*3* [10] and* mef*(E) on a MEGA element (macrolide efflux genetic assembly) [11]. The prevalence of isolates carrying both* mef* and* erm*(B) has reportedly increased. Recently two new composite elements of the Tn*916* family, containing* tet*(M) plus MEGA (Tn*2009*) and* tet*(M),* erm*(B), and MEGA (Tn*2010*) have been described [9, 12–15].

Resistance to chloramphenicol in* S. pneumoniae* is due to acetylation of the antibiotic by chloramphenicol acetyltransferase (CAT), encoded by* cat* gene, which is carried on the Tn*5253-*like, a composite structure made up of two independent conjugative transposons, when Tn*916*-like* tet*(M)-carrying element designated Tn*5251* was inserted within the Tn*5252* element that carries chloramphenicol resistance [16–18].

The distribution of pneumococcal transposons and genes they harbour varies in different geographic regions. There are scanty data as far as distribution of conjugation transposons in Poland is concerned. The aim of our study was to detect antibiotic resistance determinants and conjugative transposons in the antibiotic-resistant pneumococcal strains isolated from nasopharynx of healthy children aged 3–5 attending day care centres in Lublin, Poland.

## 2. Materials and Methods

### 2.1. Bacterial Strains

A total of 138 strains were selected from the previously described collection of 342 isolates obtained from nasopharynx of healthy 311 children aged 3 to 5 attending day care centres in Lublin, Poland [19]. The inclusion criteria were phenotype of resistance to one or more of tetracycline, chloramphenicol, erythromycin, and clindamycin.

### 2.2. Determination of the Macrolide Resistance Phenotype

The tested strains were assigned to the constitutive (cMLS), the partially inducible (iMcLS), the inducible (iMLS), or the efflux-mediated (M) macrolide resistance phenotype on the basis of the triple-disk (erythromycin plus clindamycin and rokitamycin) test, as described previously [5].

### 2.3. Amplification Experiments and Gene Detection

Bacterial genomic DNAs were prepared with Genomic Mini Kit (A&A Biotechnology, Gdynia, Poland) and were used as templates for PCR. Macrolide resistance genes* erm*(B) and* mefA/E* were detected by PCR using the primers and conditions described previously [20]. The PCR product of the* mefA/E* gene was digested with BamHI (Fermentas) to differentiate between the* mef*(A) and* mef*(E) gene subclasses [21]. PCR amplification was used to detect *cat*_*pC*194_ gene related to chloramphenicol resistance [22]. The Tn*916* and Tn*917* transposon-related genes (*int916, xis916, tnpA*, and* tnpR,* O13-O14), the tetracycline resistance gene,* tet*(M), and the promoter of the* aph3-III* gene were detected by PCR using the primers and conditions described previously [23, 24]. PCR with primer pair J12/J11 which amplified the region* orf20* to* orf19* was used to distinguish among Tn*3872*, Tn*6002,* and Tn*6003*/Tn*1545*, which yield amplicons of 0.8 kb, 3.7 kb, and 7.9 kb, respectively [23]. An* erm*(B)/*tet*(M) linkage was detected using the primers described previously [23]. The* mef*(E)-positive isolates were analyzed for the presence of Tn*2009*-like element by PCR with primers MEF4 and O14 [13, 25]. REDTaq ReadyMix (Sigma-Aldrich) was used in standard PCR and Long PCR Enzyme Mix (Thermo-Scientific) was used in reaction expected to yield PCR products exceeding 3 kb in size.

The resistance gene combination related to the different presumed transposons was Tn*6002* (*erm*(B),* tet*(M),* int*916,* xis*916, and O13-O14), Tn*3872* (*erm*(B),* tet*(M),* tnpA*,* tnpR*,* int*916, and* xis*916), Tn*6003* or Tn*1545* (*erm*(B),* tet*(M),* int*916,* xis*916, and* aph3-III*), Tn*2010* (*erm*(B),* tet*(M),* int*916,* xis*916,* mef*(E), and MEF4-O14), and Tn*2017* (*erm*(B),* tet*(M),* int*916,* xis*916,* mef*(E), MEF4-O14,* tnpA* and* tnpR*). Tn*5252* was detected by PCR of its transposase gene,* int5252*, and excisionase* xis5252* [26]. For the detection of Tn*5253*, the region of the right junction between Tn*5251* and Tn*5252* was analyzed [27]. Primers for the detection of ICESp23FST81 have been described elsewhere [26].

## 3. Results

The 138 pneumococcal isolates were characterized for genotypic attributes. Serotyping and antibiotic resistance patterns of these strains were determined previously [19]. Among the tested strains, 77 (55.8%), 35 (25.4%), 23 (18.0%), 2 (1.4%), and 1 (0.7%) belonged to serotypes 19F, 6B, 23F, 23A, and 14, respectively (Table 1). All of the strains were resistant to tetracycline (Te) but showed various resistance to other antimicrobials. A total of 71.7% strains were additionally resistant to erythromycin (E) and clindamycin (Cc) while 73.9% strains were resistant to chloramphenicol (C) as well. On the basis of the erythromycin-clindamycin-rokitamycin triple-disk test, 82 of the 99 strains resistant to erythromycin and clindamycin were assigned to the cMLS phenotype, 17 were assigned to the iMcLS phenotype, and none was assigned to the iMLS phenotype or to the M phenotype. All strains with iMcLS phenotype belonged to 6B serotype.

### 3.1. Detection of Resistance Genes

All of the strains possessed the* tet*(M) gene. All cMLS and iMcLS isolates had the* erm*(B) gene. The* mef*(E) gene, but not* mef*(A), was also found in 8 isolates with the* erm*(B) gene: 1 of the iMcLS phenotype and 7 of the cMLS phenotype. None of the strains, positive for the promoter of the* aph3-III* gene, were found. Each of the strains with resistance to chloramphenicol was positive for the *cat*_*pC*194_ gene.

### 3.2. Transposon Distribution

Table 2 shows the PCR detection of the relevant genetic determinants of resistance genes and transposons. All of the tested strains were positive for the genes coded integrase and excisionase Tn*916*, which indicated presence of elements of the Tn*916* family. For the further analysis of the* tet*(M)-carrying transposons, the strains were split into four phenotypic groups with respect to their resistance patterns, namely, Te, TeC, ECcTe, and ECcTeC. Among 16 Te isolates, in addition to integrase and excisionase genes, the PCR product of O13-O14 fragment was positive, which indicated an intact structure of the Tn916-like transposons behind the* tet*(M) gene (*orf*9) [25]. The size of* orf20-orf19* amplicon obtained in this group of strains was 0.8 kb. Seventeen isolates out of ECcTe group and 66 isolates out of ECcTeC group most probably carried the Tn*6002* transposon, as suggested by the positive O13-O14 PCR result, amplification of the* erm*(B) gene, and its connection with* tet*(M) gene as well as the size of* orf20-orf19* amplicon (3.6 kb). Additionally, 4 of ECcTeC isolates and 4 of ECcTe isolates were positive in amplification of the* mef* gene. MEGA element included in Tn*6002*, creating Tn*2010*, was suspected in 7 of them, as suggested by the positive PCR fragment with primers MEF4 and O14, and the last one was positive in Tn*917* genes (*tnp*A/*tnp*R) and negative in O13-O14 PCR product, suggesting Tn*2017* element. The vast majority of the 78 isolates from ECcTeC group produced in PCR the amplicons of integrase and excisionase genes specific for the* cat* gene-containing Tn*5252*. In six ECcTeC strains the Tn916-like and the Tn5252-like elements formed a Tn*5253*-like structure, as revealed by PCR product of the junction region [16]. Two of ECcTeC strains were found to contain the Tn3872-like transposon, being positive by the PCR products for* tnp*A+*tnp*R and linkage between* tet*(M) and* erm*(B).* Orf20-orf19* amplicon obtained from this group of strains was found to be 0.8 kb in size. Two isolates from ECcTeC group and 23 isolates of the TeC group were negative in integrase and excisionase specific for* Tn5252*, but these isolates possessed transposase specific for* cat*-containing ICESp23FST81 element.

**Figure 1:**
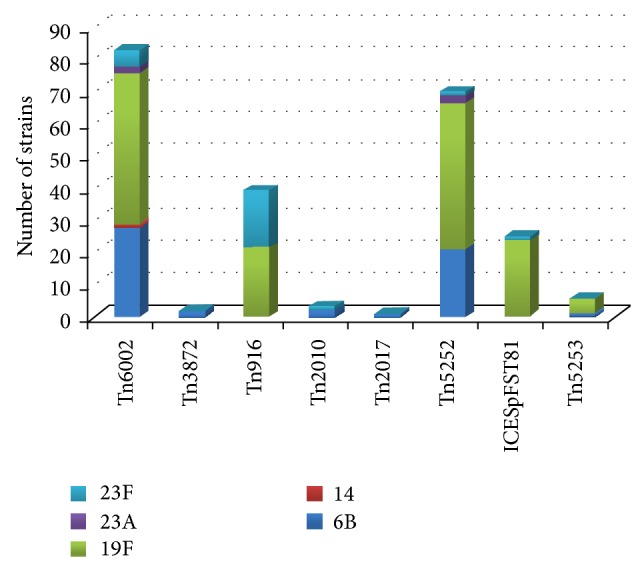
Transposon distribution among pneumococcal serotype of 138* S. pneumoniae* nasopharyngeal isolates from healthy young children.

Figure 1 shows the distribution of presumable transposons among serotypes of the tested strains.

## 4. Discussion

Antibiotic resistance in* S. pneumoniae* is often carried by the mobile genetic elements that reside in the chromosome, such as conjugative transposons. According to our knowledge only Izdebski et al. [27] showed the data concerning analysis of* tet*(M)-carrying mobile elements among clinical isolates obtained from respiratory tract infections in Poland. In our study, we assessed distribution of resistance genes as well as presumable conjugative transposons in 138 resistant or multiresistant nasopharyngeal strains of* S. pneumoniae* obtained from healthy preschool children attending day care centres.

Macrolide and tetracycline resistance of pneumococci has increased in European countries, for example, Italy, France, and Spain, as well as in North America [28]. Poland belongs to a part of Europe with a high level of antibiotic consumption and above 20% rate of tetracycline and macrolide resistance among the pneumococcal isolates [29]. Resistance to macrolides associated with MLS_B_ phenotype is predominant in most European countries, whereas the M phenotype predominates in North America, England, and Germany [30]. Our study confirmed that macrolide-resistant pneumococci in Poland are associated mainly with the MLS_B_ phenotype, confirmed by the presence of* erm*(B) gene in the tested strains, as previously reported [27]. None of the tested strains possessed M phenotype as well as* mef* gene as a sole determinant of erythromycin resistance. Previous studies by Izdebski et al. [27] revealed among clinical pneumococcal strains in Poland only 4.7% macrolide-resistant strains which possessed* mef*(E) instead of* erm*(B).

A worldwide emergence of pneumococci harboring both* erm*(B) and* mef*(E) genes, with the global prevalence of 16.4% among macrolide-resistant isolates, has been described [31]. The recent data from China have reported dramatically high proportion (62.9%) of pneumococcal isolates with both* erm*(B) and* mefA/E* genes [32]. In the present study, we found only 8.1% macrolide-resistant isolates that harboured both genes and they were observed in 6B serotype, mostly.

The analysis of the mobile elements presented in this paper revealed their remarkable diversity in the population studied. Presence of Tn*916* which is the prototype of the Tn916 family was observed in all tetracycline-resistant but macrolide- and chloramphenicol-susceptible strains with 23F serotype, comprising 11.6% of tested strains. The number of such strains was much lower than that described previously—38.8% [27].

There are some geographical differences in the prevalence and spread of transposons carried* erm*(B) gene in pneumococci. In Italy and Spain, the most frequently reported transposons in* S. pneumoniae* are Tn*6002* and Tn*3872*, whereas in Japan Tn*917* was found to be the most common [14, 23, 24, 33]. Tn*3872* is a composite, mobile element resulting from the insertion of the* erm*-containing Tn*917* transposon into Tn*916* [8, 23]. Our study showed that Tn*6002* was more common among the erythromycin-resistant isolates, which accounts for 83.8% and Tn*3872*-related elements were found in only 3 of* erm*(B)-carrying* S. pneumoniae* strains with 6B serotype (3%), including one of them which is likely to possess larger mobile element, Tn*2017*. A study performed in China in 2010 on 135 macrolide-resistant pneumococci from nasal swabs of children revealed the high prevalence of Tn*6002*-like elements (56.3% of isolates) and Tn*3872* (5.2%) [34]. This observation is comparable to other authors' data [14, 23, 33].

Kanamycin resistance is related to Tn*1545*, a Tn*916* family element that acquired* erm*(B) and* aph3*'III genes. A low prevalence of kanamycin resistance detected among the erythromycin- and tetracycline-resistant pneumococci was observed by other authors [8, 14, 27, 34] while our data showed lack of* aph3*-III gene among the studied strains. Besides, in the present study there were no isolates possessing all genetic markers pointed to the presence of Tn*1545*/Tn*6003* element.

Among the macrolide-resistant* mef*(E)-carrying strains, the presence of genetic element carrying a MEGA element such as Tn*2009*, Tn*2010*, and Tn*2017* would be expected [13, 15]. In this study, strains harbouring both* mef*(E) and* erm*(B) genes were detected; therefore the Tn*2010* and Tn*2017* transposons were suggested. Tn*2010* was first detected in an Italian isolate [13] and then it was detected in the different studies with various frequencies (2–28.9%) [14, 34]. The presence of Tn*2010* in 72.2% of macrolide-resistant isolates from China was detected and the CC271 strains carrying the Tn*2010* element expressing the high-level resistance to erythromycin were predominant in China [35].

All of the studied isolates which were resistant to chloramphenicol proved to harbour *cat*_C194_ carried by Tn*5253*-like element. These composite elements demonstrate substantial heterogeneity results from at least two levels of variability, in the Tn*5252*-like and in the Tn916-like transposons [26]. Two variants of the Tn*5252*-related transposon were distinguished depending on the presence of the* int*5252 and* xis*5252 or *int*_Sp23FST81_ gene. Interestingly, all of the strains with TeC resistance pattern and 19F or 23F serotype possessed elements positive for *int*_Sp23FST81_. This can be the proof of clonal spreading of these strains in children population. The presence of Tn*5253*-family and ICESp23FST81-family elements has been investigated in clinical isolates of* S. pneumoniae* and proven to be frequent, especially among multidrug resistant strains [26, 36].

Both Tn*916* and another transposon from a number of Tn*916* family pneumococcal relatives carrying a variety of insertion containing erythromycin resistance genes such as* erm*(B) or* mef*(E) or both in addition to the* tet*(M) tetracycline resistance determinant typical for the family [24, 37] can be found as independent elements and inserted into Tn*5252*-related transposons to form a Tn*5253*-like composite element [26]. It is possible that in most of studied strains,* tet*(M) and* erm*(B) reside in Tn916-like elements, whereas* cat* gene was located in separate transposons. Out of 101 chloramphenicol-resistant strains, in which* cat*-containing transposon was detected, only in 5.9% isolates an association with* tet*(M) was confirmed, as in the case of Tn*5253*. However, the possibility that other Tn916-like elements were inserted into Tn*5252*-related transposon could not be excluded and additional experiments should be conducted with suitable primer pairs to seek a linkage with the associated Tn916-like transposon.

The 138 resistant/multiresistant* S. pneumoniae* strains tested were distributed into 5 serotypes and 37 BOX PCR types [19]. These findings, in addition to the genotypic differences found in this study, indicate that the studied strains were partially related to the place of isolation (day care center), regarding isolates with 19F ECcTeC phenotype especially. This suggests a spread of resistance due to clonal dissemination mainly. Children in day care centres due to close contact with each other are more frequently at risk of respiratory tract infections and antimicrobial consumption. Their nasopharynx can be colonized by pneumococci and other types of bacteria for up to a few months facilitating transfer of genetic material between bacterial strains [9, 38]. Children nasopharynx is therefore a likely place for antimicrobial resistance to emerge, and resistance rates can increase fast. The epidemiological relationship between strains colonizing healthy children and strains causing pneumococcal infections has been documented before, so surveillance studies of pneumococcal carriage allow monitoring strains circulating in community, including their antibiotic resistance.

## 5. Conclusions

The high prevalence of macrolide, tetracycline, and chloramphenicol resistance among* S. pneumoniae* strains was mainly due to clonal dissemination of multiresistant strains in the children populations. However, owing to phenotypic and genotypic differences observed in tested strains, horizontal spread of the Tn*916* family of transposons, which can be found as independent elements as well as inserted into Tn*5252*-related transposons to form a Tn*5253*-like composite element, was also feasible.

## Figures and Tables

**Table 1:** Resistance patterns and serotypes of 138 *S. pneumoniae* nasopharyngeal isolates from healthy young children.

Resistance pattern	Serotypes (number of strains)
Te	23F (16)
TeC	19F (22), 23F (1)
ECcTe	6B (13), 14 (1), 19F (2), 23F (5)
ECcTeC	6B (22), 19F (53), 23A (2), 23F (1)

E: erythromycin, Cc: clindamycin, Te: tetracycline, and C: chloramphenicol.

**Table 2:** Antibiotic resistance and transposon genes identified among 138 *S. pneumoniae* nasopharyngeal isolates from healthy young children.

Serotype (number of strains)	Resistance pattern	Genes detected by PCR		Presumptive transposons	
		Resistance genes	Transposon genes	Tn916 family element	Tn5252-like element
23F (16)	Te	*tet*(M)	*int/xis*916	Tn*916 *	ICE*Sp*23FST81-like
19F (22), 23F (1)	TeC	*tet*(M),* cat*_pC194_	*int/xis*916, *intICE *	Tn*916 *	
6B (10), 14 (1), 19F (2), 23F (4)	ECcTe	*tet*(M), *erm*(B)	*int/xis*916	Tn*6002 *	
6B (2), 23F (1)	ECcTe	*tet*(M), *erm*(B), *mef*(E)	*int/xis*916	Tn*2010 *	
6B (1)	ECcTe	*tet*(M), *erm*(B), *mef*(E)	*int/xis*916, *tnp*A/*tnp*R	Tn*2017 *	
6B (18), 19F (43), 23A (2), 23F (1)	ECcTeC	*tet*(M), *erm*(B),* cat*_pC194_	*int/xis*916,* int*/*xis*Tn5252	Tn*6002 *	Tn*5252 *
6B (1), 19F (5)	ECcTeC	*tet*(M), *erm*(B),* cat*_pC194_	*int/xis*916,* int*/*xis*Tn5252, *jun*Tn5253	Tn*6002 *	Tn*5253*-like
6B (1), 19F (3)	ECcTeC	*tet*(M), *erm*(B), *mef*(E),* cat*_pC194_	*int/xis*916,* int*/*xis*Tn5252	Tn*2010 *	Tn*5252 *
6B (2)	ECcTeC	*tet*(M), *erm*(B),* cat*_pC194_	*int/xis*916, *tnp*A/*tnp*R, *int*/*xis*Tn5252	Tn*3872 *	Tn*5252 *
19F (2)	ECcTeC	*tet*(M), *erm*(B),* cat*_pC194_	*int/xis*916,* intICE *	Tn*6002 *	ICE*Sp*23FST81-like

E: erythromycin, Cc: clindamycin, Te: tetracycline, and C: chloramphenicol.
